# Three-dimensional kinematic analysis of the pectoral girdle during upside-down locomotion of two-toed sloths (*Choloepus didactylus*, Linné 1758)

**DOI:** 10.1186/1742-9994-7-21

**Published:** 2010-07-12

**Authors:** John A Nyakatura, Martin S Fischer

**Affiliations:** 1 Institut für Spezielle Zoologie und Evolutionsbiologie mit Phyletischem Museum, Friedrich-Schiller-Universität, Erbertstrasse 1, 07743 Jena, Germany

## Abstract

**Background:**

Theria (marsupials and placental mammals) are characterized by a highly mobile pectoral girdle in which the scapula has been shown to be an important propulsive element during locomotion. Shoulder function and kinematics are highly conservative during locomotion within quadrupedal therian mammals. In order to gain insight into the functional morphology and evolution of the pectoral girdle of the two-toed sloth we here analyze the anatomy and the three-dimensional (3D) pattern of shoulder kinematics during quadrupedal suspensory ('upside-down') locomotion.

**Methods:**

We use scientific rotoscoping, a new, non-invasive, markerless approach for x-ray reconstruction of moving morphology (XROMM), to quantify *in vivo *the 3D movements of all constituent skeletal elements of the shoulder girdle. Additionally we use histologic staining to analyze the configuration of the sterno-clavicular articulation (SCA).

**Results:**

Despite the inverse orientation of the body towards gravity, sloths display a 3D kinematic pattern and an orientation of the scapula relative to the thorax similar to pronograde claviculate mammalian species that differs from that of aclaviculate as well as brachiating mammals. Reduction of the relative length of the scapula alters its displacing effect on limb excursions. The configuration of the SCA maximizes mobility at this joint and demonstrates a tensile loading regime between thorax and limbs.

**Conclusions:**

The morphological characteristics of the scapula and the SCA allow maximal mobility of the forelimb to facilitate effective locomotion within a discontinuous habitat. These evolutionary changes associated with the adoption of the suspensory posture emphasized humeral influence on forelimb motion, but allowed the retention of the plesiomorphic 3D kinematic pattern.

## Background

In therian mammals (i.e., marsupials and placental mammals) the shoulder girdle is mobilized when compared to monotremes and other amniotes [1]. The coracoids are reduced and the scapula became an important propulsive element in the forelimb [2-4]. It has been shown that both forelimb kinematics [5] and the relative proportions of propulsive elements are rather conservative in quadrupedal therian mammals [6] - observations that have been attributed to biomechanical constraints of limb configuration (see review in [7]).

In 1935 the functional anatomist Ruth Miller wrote "of all mammals the sloths have probably the strangest mode of progression" [8]. Due to their characteristic 'upside-down' posture and locomotion these animals represent interesting 'natural experiments' to study functional implications of the inverse body orientation with regard to the force of gravity. Unlike in 'normal', pronograde locomotion, a tensile loading regime acts within the limbs rather than a compressive one [9], and flexors are required to counteract the gravity-induced extension of the limbs [10]. Many morphological peculiarities of the limbs were stringently interpreted as adaptations to facilitate the upside-down posture and locomotion of sloths. For example, the anatomy of the main flexor muscles of the limbs (m. brachioradialis and m. biceps femoris) have largely advantageous moment arms on the joints they span to flex the forelimb against gravity-induced extension [10].

Earlier studies on sloth functional anatomy concentrated on the skeletal and muscular adaptations of the distal limbs to suspensory posture [8,10-13] or more general aspects of the overall locomotor pattern [14-16], and there is considerably less data available for the interpretation of possible functional aspects of peculiarities of the proximal limbs and their connection to the axial skeleton, i.e., the pectoral and pelvic girdles. The gravity vector has fundamental relevance for the connection between the limbs and the thorax. Extrinsic forelimb musculature acting on the pectoral girdle not only protracts and retracts the forelimb but also functions to suspend the weight of the thorax in mammals [17,18].

In claviculate mammals the clavicle is the only remaining skeletal connection of the forelimbs to the thorax [1]. Although in both extant sloth genera a clavicle is developed, it has an unusual articulation to the sternum (manubrium sterni), because the articulating faces are lacking [e.g., [19]]. The sterno-clavicular articulation (SCA) is described as either cartilaginous [19] or ligamentous [8,10]. Whatever the configuration of the SCA, a functional significance is implied, as authors point out the extraordinary mobility of the clavicle at this joint [8,10,19].

**Figure 1:**
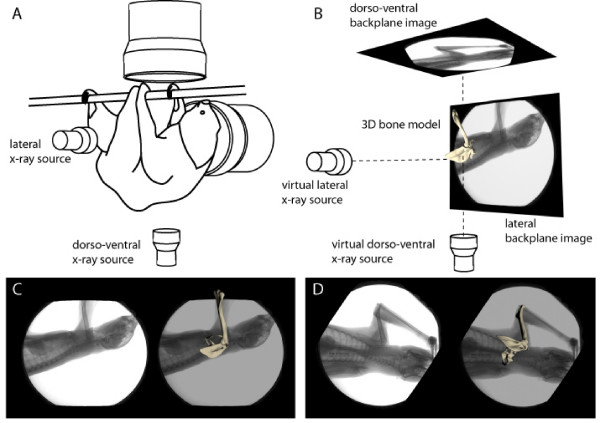
**Scientific rotoscoping **[20]. The experimental setup for synchronous high-speed x-ray video recording (A) is virtually re-created within animation software (B). X-ray videos are loaded into the backplane as image sequence planes. A 3D bone model is positioned to match the x-ray shadow in both the lateral (C) and dorso-ventral (D) backplane for the entire image sequence. Six DOF (degrees of freedom, i.e., translations and rotations about anatomically defined coordinate systems - see methods) are exported for all constituent skeletal elements of the shoulder girdle.

We present a precise reconstruction of the three-dimensional (3D) *in vivo *motion of skeletal structures of the pectoral girdle to gain insight into the function of the pectoral girdle in two-toed sloths (Xenarthra: *Choloepus didactylus*, Linné 1758) and the evolutionary changes of shoulder function associated with the adoption of the suspensory quadrupedal posture and locomotion. The data were obtained using 'scientific rotoscoping' (SR) [20], a markerless, non-invasive approach for x-ray reconstruction of moving morphology (XROMM) [21]. This technique combines synchronous biplane high-speed x-ray video and x-ray computed tomography scans to visualize and measure three-dimensional motions of the pectoral girdle usually hidden under integument, muscles and other tissue (Fig. 1). Thanks to this new approach we are able to report six degrees of freedom (DOF) data for all constituent skeletal elements of the pectoral girdle in sloths and test for the significance of different aspects of the movement of individual skeletal elements in 'virtual experiments'. Additionally, the SCA is examined using histochemical methods to aid functional interpretation of its configuration. Morphological and kinematic changes that are associated with the adoption of the suspensory quadrupedal locomotion during the evolution of modern sloths will be discussed. Analysis of the functional morphology of the pectoral girdle in sloths may yield insights into constraints and flexibility of the mammalian shoulder girdle to functional demands.

## Methods

### Anatomical investigation

No sloths were sacrificed for this study. All procedures and animal care were carried out in compliance with the animal welfare regulations of the state of Thuringia, Germany (Reg.-Nr.: 02-08/04). Two frozen specimens of adult female *C. didactylus *from zoos in Paris, France, and Dresden, Germany were donated to us. The skin was removed and the subjects were formalin fixed. Prior to histological staining, tissue from the sterno-clavicular articulation (SCA) was prepared from one of the donated specimens by first removing all muscular connections from the clavicle and manubrium sterni and subsequently cutting through the midpoint of the clavicle and the middle of the manubrium sterni. Bone remnants were carefully removed *ex situ *and connective tissue from the articulation was embedded in paraffin before being sectioned with a Microm™ HM360 microtome (10 μm sections). Serial sections from medial to lateral were produced and histological properties were analyzed using differential staining methods (Hämalaun-Eosin (HE), Azan, and Masson-Goldner) on consecutive sections.

In order to rule out the possibility that we were studying morphological extremes in two animals used for the locomotion analysis, we additionally measured skeletal properties in the few available adult (fused epiphyses of long bones) *C. didactylus *individuals obtained from German museum collections (Museum f. Naturkunde, Berlin; Zoologisches Museum, Hamburg). We found our experimental subjects to lie mostly within one standard deviation of the anatomical variability of the small sample (Table 1).

**Table 1:** Comparative measurements of pectoral girdle elements.

Specimen	scapula length	**spina scapulae l**.	humerus length	clavicle length	ulna length	hand length
Museum material						
*C. didactylus *(MfN 102636)	8.98	5.74	14.06	4.64	17.02	x
*C. didactylus *(ZMH 1507)	7.32	5.18	12.94	3.80	13.91	8.81
*C. didactylus *(ZMH 1506)	7.25	4.45	11.75	3.21	14.42	x
Dissected specimens						
*C. didactylus*	8.68	5.73	15.7	4.52	19.4	10.01
*C. didactylus*	7.71	5.24	14.58	4.2	17.38	9.2
Study animals*						
*C. didactylus *(male)	7.7	5.0	13.2	3.6	15.2	9.0
*C. didactylus *[female)	8.2	5.8	15.9	4.6	19.4	9.8
Mean (± s.d.)	7.98 (± 0.67)	5.31 (± 0.49)	14.02 (± 1.51)	4.08 (± 0.56)	16.68 (± 2.25)	9.37 (± 0.52)

*: measurements of XROMM study animals retrieved from x-ray images.

Scapula length is measured as distance from angulus inferior to acromio-clavicular joint; spina scapulae length is measured along spina scapulae from vertebral border to glenoid cavity. Hand length is measured from wrist joint to distal interphalangeal joint. All values are reported in cm. MfN: Museum für Naturkunde Berlin; ZMH: Zoologisches Museum Hamburg; x: missing data due to incompleteness of museum material.

### Biplane high-speed x-ray motion analysis: experimental setup

X-ray videos of two adult *C. didactylus *of different weights and sex were recorded. Neither the female (10.6 kg, 87 cm body length measured from tip of nose to ischium) nor the male (6.5 kg, 78 cm) displayed any physical and behavioral peculiarities. X-rays were taken synchronously from the dorso-ventral and latero-lateral projections during steady-state locomotion (Fig. 1). Subjects were trained to move along a motor-driven 'treadpole' (4000 × 40 mm), which permitted the x-ray recording of several consecutive strides in a trial. Stride cycles were treated as independent events. Between 0.2 m/sec and 0.3 m/sec locomotion of two-toed sloths has been shown to be relatively uniform [16]. All slower and faster trials were discarded for the present study and only strides with symmetry values (i.e., the elapsed share of a given limb cycle at touch down of the contralateral limb) between 0.4 and 0.6 were analyzed for the sake of uniformity. We used the Student's t-test for independent samples (analyses carried out in SPSS™ 12.0) to test whether both individuals differed significantly in regard of gait parameters in different strides (sample size n = 14 and n = 18, respectively; Table 2). Intra-individual variability of gait parameters appeared to be so distinct that inter-individual differences carried no weight. Based on the observation of statistically insignificant differences between subjects in stride length, swing phase duration and contact phase duration, as well as scapula touch down angle projected to the parasagittal plane (i.e., the 2D angle obtained from the latero-lateral projection) and the qualitative comparison of inter- and intra-individual variability (Table 2), stride cycles from both experimental individuals were subsequently pooled. 10 steady-state stride cycles in each study subject were analyzed. All trials were time normalized to 50 points over the contact phase and swing phase, respectively, to facilitate compilation of multiple trials so that the mean and standard deviation of the kinematic curves could be determined.

**Table 2:** Intra-individual differences and inter-individual variability of gait parameters.

	Individual 1 (mean ± s.d.)	Individual 2 (mean ± s.d.)	Comparison of intra-individual variability (p-value*)	Both individuals pooled (mean ± s.d.)
Stride length (in cm)	57.4 ± 4.8 (n = 18)	60.4 ± 4.9 (n = 14)	0.098 n.s.	58.9 ± 5.0 (n = 32)
Forelimb swing phase duration (in sec)	0.81 ± 0.1 (n = 18)	0.88 ± 0.3 (n = 14)	0.316 n.s.	0.84 ± 0.2 (n = 32)
Forelimb contact phase duration (in sec)	1.47 ± 0.2 (n = 18)	1.52 ± 0.2 (n = 14)	0.541 n.s.	1.50 ± 0.2 (n = 32)
Scapula touch down angle (in degree)	43.7 ± 5.5 (n = 10)	40.4 ± 4.9 (n = 10)	0.174 n.s.	42.0 ± 5.3 (n = 20)

*: significant differences when p ≤ 0.05, n.s.: not significant.

The Student's t-test for independent samples was used to test for significant differences in intra-individual variability of gait parameters.

Both 40 cm diameter image intensifiers were equipped with a Visario Speedcam™ (Weinberger GmbH, Erlangen, Germany) and recorded at a resolution of 1.536 × 1.024 pixels and a speed of 300 frames per second (fps). A calibration object (20 × 12 × 12 cm) with metal beads inserted at 1 cm distances was used to calibrate the 3D space covered by both x-ray devices for subsequent analysis using 11 parameter direct linear transformation (DLT; necessary Matlab™ files available at http://www.xromm.org) [21].

### X-ray reconstruction of moving morphology (XROMM)

Scans of disarticulated skeletal elements were taken using a GE Lightspeed™ 16 CT scanner at the Zentralklinik, Bad Berka, Germany, at 120 kV and 150 mA. Voxel size of the scan was 0.47815 mm with a slice thickness of 0.625 mm. To reconstruct bone models raw data was surface rendered in Imaris™ 6.4 and converted into .obj file format using customized software (by H. Stark available at http://www.stark-jena.de). Models were imported into Maya™ 8.0 and hierarchically connected via virtual joints to form a digital marionette [20]. In order to avoid possible harm of the valuable zoo animals from anesthesia a different skeleton was scanned and then scaled to match the size of the experimental subjects by using the scale tool in Maya™ and the calibrated x-ray references in the backplane.

Distortion of all x-ray recordings was corrected using a reference grid [see [21]]. In Maya™ virtual dorso-ventral and latero-lateral cameras were created and their relative position in virtual 3D space calibrated so that they imitated the actual x-ray sources (necessary Matlab™ and Maya™ embedded language files available at http://www.xromm.org) (Fig. 1). The un-distorted x-ray image sequences from both projections were put in the backplane of the recreated x-ray cameras.

**Figure 2:**
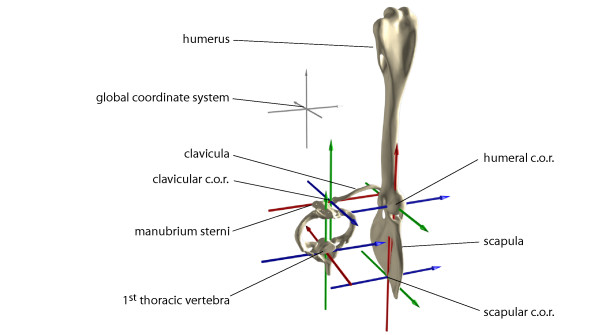
**Anatomical coordinate systems and zero-positions of rotations are used to quantify three-dimensional kinematics of the pectoral girdle**. The anatomical coordinate systems were placed in the center of rotation (c.o.r.) of the proximally adjacent joint (in case of 1^st ^thoracic vertebra into the center of the vertebral body; in case of scapula we approximated the instantaneous c.o.r. at the vertebral border of the scapula at the extension of the spina scapulae). *X*-axes (red) were set to represent the long axis of elements. *Z*-axes (blue) were oriented to represent the most distinct motion of the bone of interest. *Y*-axes (green) were orthogonal to the other two axes. For zero-points of rotations the anatomical axes were aligned according to the global coordinate system (unnatural pose). Motions of hierarchically higher elements have displacing effect for all lower ranked elements, i.e., motions of humerus are reported relative to scapula, scapular and clavicular motion relative to 1^st ^thoracic vertebra, 1^st ^thoracic vertebra motion relative to global reference.

Motions of the modeled skeletal elements forming the digital marionette are reported relative to hierarchically higher ordered elements (Table 3). Right handed anatomical coordinate systems were implemented at the center of rotation of each element (Fig. 2). Translations were set to zero at the instant of touch down.

**Table 3:** Anatomical coordinate systems used for the kinematic analysis.

Joint/element (hierarchy)	Anatomical meaning of rotation about axis	Zero-point for rotations
Global coordinate system (top)		
*x-axis*	-	-
*y-axis*	-	-
*z-axis*	-	-
1^st ^thoracic vertebra (1^st ^order)		
*x-axis*	Long axis rotation of vertebral column (roll)	Aligned to global *x*
*y-axis*	Lateral undulation of vertebral column (yaw, +: undulation to the right)	Aligned to global *y*
*z-axis*	Pitch of vertebral column (+: decrease of head-support distance)	Aligned to global *z*
Scapular center of rotation/scapula (2^nd ^order)		
*x-axis*	Inward (+) /outward (-) rotation about long axis of scapula	Scapula is not rotated (long axis of scapula parallel to thoracic *y*-axis)
*y-axis*	Abduction (-) /adduction (+) of scapula (yaw)	Scapula is not abducted
*z-axis*	Protraction (-) /retraction (+) of scapula (pitch)	Scapula is vertical (in perfect dorso-ventral orientation)
Glenohumeral joint/humerus (3^rd ^order)		
*x-axis*	Long axis rotation of humerus (roll, +: outward rotation)	Humerus is not rotated (epicondyles aligned in frontal plane)
*y-axis*	Humeral abduction (-) /adduction (+) from scapular plane (yaw)	Humerus is in scapular plane
*z-axis*	Humeral protraction (-) /retraction (+) (flexion in glenohumeral joint, pitch)	Humerus is orientated vertical (long axis parallel to scapula long axis)
Sterno-clavicular joint/clavicle (2^nd ^order)		
*x-axis*	Rotation about long axis of clavicle (+: caudal rotation)	The curvature of the clavicle is pointing ventral
*y-axis*	Anterior (+) /posterior (-) displacement of acromio-clavicular joint relative to manubrium sterni	Clavicle is pointing lateral and forms 90° angle to long axis and *y*-axis of the 1^st ^thoracic vertebra
*z-axis*	Dorso (-) /ventral (+) displacements of acromio-clavicular joint relative to manubrium sterni	Clavicle is pointing lateral and forms 90° angle to long axis and *y*-axis of the 1^st ^thoracic vertebra

Right handed coordinate systems are used for each joint with the *x*-axis oriented along the long axis of the bone of interest, *z*-axis always oriented to represent the most distinct motion of this bone, and *y-axis *orthogonal to both other axes (see Fig. 2). The anatomical coordinate systems are placed directly where the motion takes place, i.e. in the joint, and are fixed to the proximally adjoining bone. This means that motion of the bone of interest is reported relative to the proximally adjoining bone. Motion of the 1^st ^order hierarchy bone (1^st ^thoracic vertebra) is reported relative to a global coordinate system with positive *x *in direction of movement, positive *y *towards dorso-ventral image intensifier (ventral to the animal), and positive *z *to animal's left. + = positive rotation about respective axis; - = negative rotation about respective axis.

During SR the digital marionette was then positioned to match the x-ray shadow of both projections for every fifths frame. After a trial had been completed the three rotations representing the movement of a bone relative to the higher ordered skeletal element were exported into Microsoft™ Excel.

In SR, accuracy and repeatability depends on many factors, including the quality of calibration, the visibility of skeletal structures on the x-ray references, the temporal resolution of reference x-ray videos, and on the effort of the investigator. Due to the unequal shape and thickness of the structures studied, there is not one value that can represent the accuracy of all measurements. General accuracy for optimal conditions was measured by comparing the known opening of a vernier caliper (150 mm) to the measured opening following the approach used in this study. We determined an opening of 150.704 mm; i.e., a deviation of less then one millimeter. To assess repeatability we determined the scapular lift off position of a single trial on ten consecutive days of data analysis and found only small deviations (mean ± s.d.): lift off frame 708.3 ± 0.95 (i.e., s.d. is less than 1 frame or 1/300 sec); trans × -0.43 cm (± 0.13); trans y -0.83 cm (± 0.12); trans z 0.17 cm (± 0.12); rot × -18° (± 0.76); rot y -18° (± 0.24); rot z 38° (± 0.16). Due to these deviations we report all kinematic data rounded to the closest tenth of a cm and degree, respectively.

### Quantification of displacing effects of individual elements

One of the merits of the XROMM approach to kinematic analysis is that it allows 'virtual experiments' with the animated 3D reconstruction. Here, we quantify the displacing effects of individual elements of a joint chain by turning off its movements either by 'muting' all translations and rotations or by 'muting' only specific rotations. With XROMM it is possible, for example, to turn off abduction and adduction of a bone relative to its defined anatomical coordinate system. The displacing effect of the motion of an element can then be assessed by comparing the displacement of normal movements at the most distal point of the joint chain to the displacement of the most distal point in the joint chain obtained in the 'virtual experiment', with aspects of the motion turned off. In this study we assess the displacing effects of aspects of scapular and humeral motion on the displacement of the elbow. The displacement of the elbow is obtained relative to the first thoracic vertebra. We subsequently turned off total scapular motion (all translations and rotations), total humeral motion, scapular rotation along its long axis, scapular abduction/adduction, and humeral abduction/adduction from the scapular plane. In each of these experiments motion was turned off in the moment of touch down; i.e., initial position of the elbow is the same in each case but displacement is altered during the course of the 'virtual' contact phase and subsequent swing phase. Three dimensional trajectories of the elbow are reported and total deviations among the 'virtual experiments' can thus be compared.

## Results

### Skeletal anatomy of pectoral girdle

The thorax of *C. didactylus *has 23 to 24 ribs, of which 12 are connected to the sternum [22]. In cranial direction the thorax tapers considerably. The SCA allows virtually all degrees of freedom, when the clavicle is stripped of attaching muscles [10]. The internally curved clavicle articulates to the scapula in a small notch at the widely flared acromial process. Fused to the coracoid process, the acromion forms an arch that is extended cranially beyond the humeral head. It provides prominent attachment sites for the m. trapezius, m. subclavius, and m. deltoideus. When compared to forelimb intralimb proportions reported for large dataset of mammalian species [6], the relative length of the scapula of *C. didactylus *(expressed as percentage of sum of scapula, upper arm and lower arm length) is very short [21% instead of ca. 30%; cf. 6]. The scapula is thin and the spina scapulae does not reach the vertebral border. The caudal border is characterized by a broad attachment site for the well developed m. teres major. The gleno-humeral joint is marked by a shallow fossa glenoidalis and round humeral head. There is a considerable incongruity between both articulating surfaces.

### Histologic properties of the SCA

**Figure 3:**
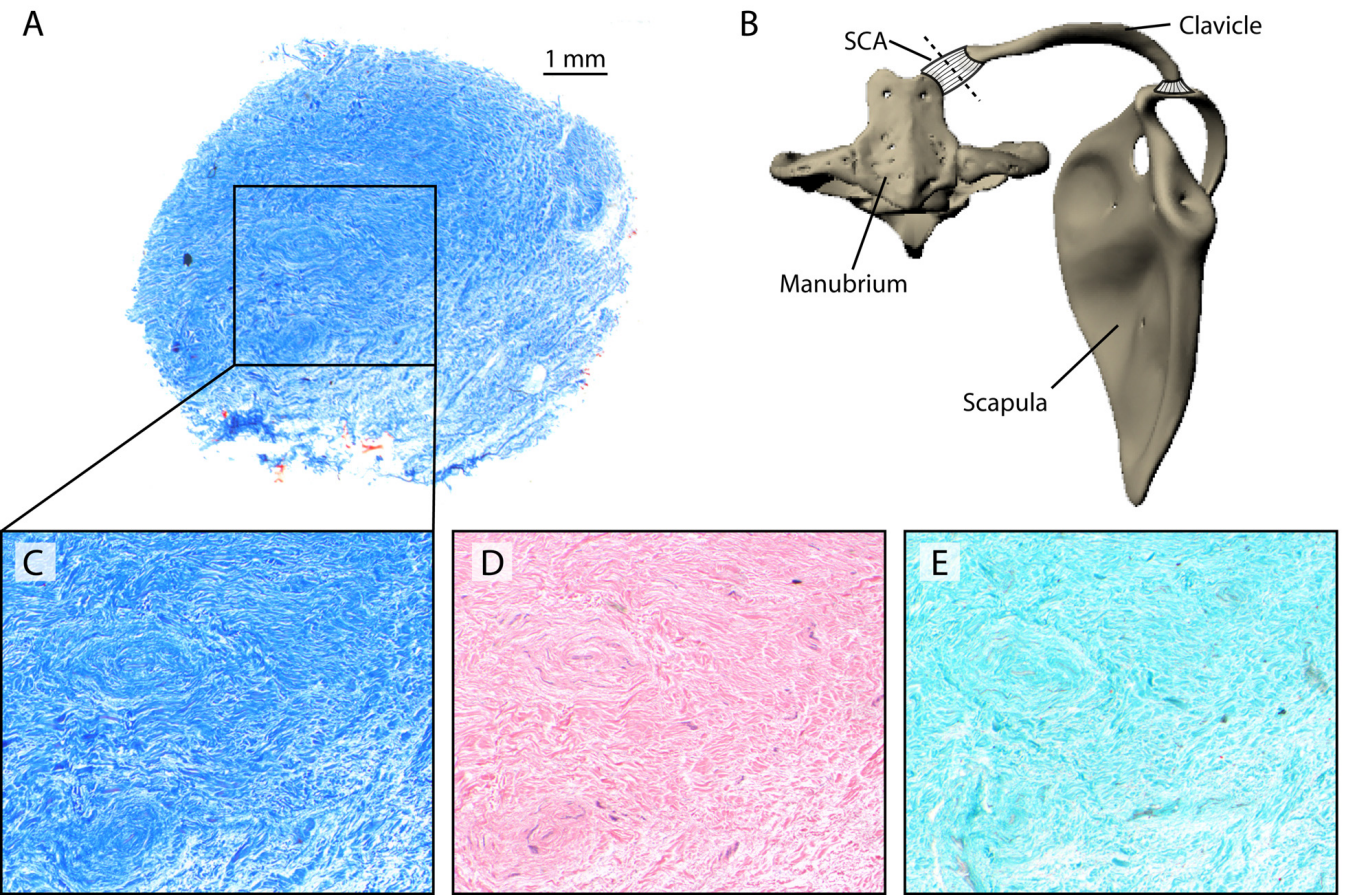
**Histological properties of the sterno-clavicular articulation (SCA)**. A: Overview of representative cross section, B: illustration of manubrium sterni (ventral aspect), clavicle, scapula, and position of the representative cross section through the SCA (dotted line), C-E enlarged insets of A stained differently on subsequent sections. Panels A and C stained with Azan, D stained with HE, E stained following Masson-Goldner protocol. The articulation comprises solely irregular fibrous connective tissue. Neither elastic fibers nor cartilage or muscle tissue are evident. The collagen fibers do not form regular parallel bundles. No synovial cavity is present.

Serial cross sections and differential staining of the SCA from proximal to distal revealed a homogenous fibrous composition of the structure (Fig 3). No elastic or reticular fibers are evident, as they would have stained orange-red in Azan staining and light green in Masson-Goldner staining. Also, there is no cartilage or fibrocartilage within the SCA, as it would have stained blue in the HE staining. Additionally, there is little intercellular substance (violet in HE staining) apart from the intercellular collagen. Thus the SCA can be identified as dense connective tissue containing irregular arranged collagen fiber bundles. There was no synovial cavity found.

### Thorax movements

**Figure 4:**
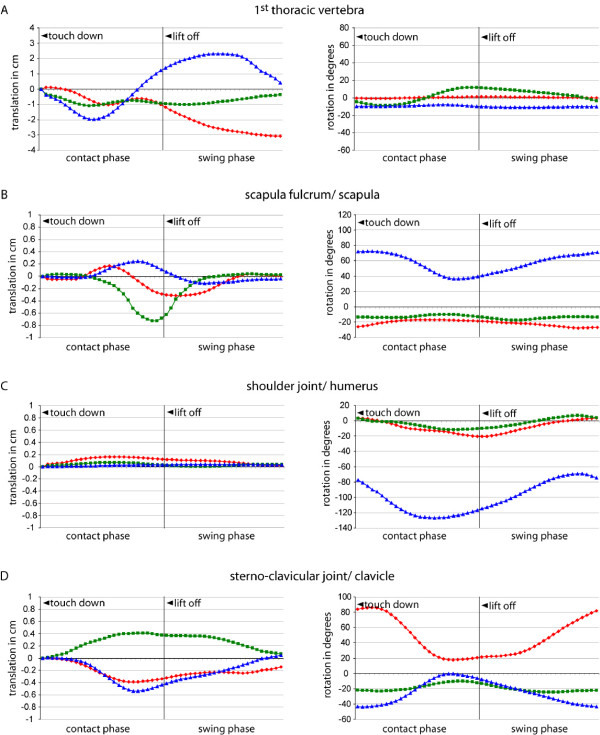
**Mean 3D kinematics of the pectoral girdle over a step cycle (n = 20)**. A: 1^st ^thoracic vertebra; B: scapular center of rotation/scapula; C: shoulder joint/humerus; D: sterno-clavicular joint/clavicle. Translations shown left, rotations right. Translations and rotations about *x*-axes are red, translations about *y*-axes are green, translations and rotations about *z*-axes are blue. The anatomical significance of these motions is detailed in Table 3 and Fig. 2.

During quadrupedal suspensory locomotion in sloths, the thorax experiences side to side displacement over the course of a step cycle (translation along *z*-axis in Fig 4A). In combination with the tapered shape of the rib cage, this movement leads to the approximate alignment of the thoracic wall with the parasagittal plane at the moment of forelimb lift off at the same body side. Rotation about the *y*-axis indicates that during contact of a forelimb the long axis of the 1^st ^thoracic vertebra first points towards the contralateral side and then rotates to face the ipsilateral body side at lift off. We documented almost no rotation about the long axis and a relatively constant pitch towards the support in the 1^st ^thoracic vertebra (Table 4).

**Table 4:** Six DOF kinematic data for the elements of the pectoral girdle during steady-state locomotion of *C. didactylus*.

	Touch down (± s.d.)	Lift off (± s.d.)	Contact amplitude	Maximal amplitude
1^st ^thoracic vertebra				
*x*-axis translation	0	-1.0 (± 2.1)	1.0	3.2
*y*-axis translation	0	-0.9 (± 0.9)	0.9	1.1
*z*-axis translation	0	1.2 (± 1.7)	1.2	4.3
*x*-axis rotation	-0.4 (± 3.5)	1.0 (± 4.0)	1.4	1.9
*y*-axis rotation	-4.6 (± 6.2)	11.5 (± 5.7)	16.2	20.5
*z*-axis rotation	-10.1 (± 4.0)	-9.8 (± 2.6)	0.3	3.0
				
Scapula center of rotation/scapula				
*x*-axis translation	0	-0.3 (± 0.2)	0.3	0.5
*y*-axis translation	0	-0.7 (± 0.1)	0.7	0.8
*z*-axis translation	0	0.1 (± 0.3)	0.1	0.4
*x*-axis rotation	-26.3 (± 8.1)	-18.6 (± 9.2)	7.7	10.7
*y*-axis rotation	-13.5 (± 3.2)	-13.1 (± 5.0)	0.4	7.6
*z*-axis rotation	71.7 (± 12.9)	39.3 (± 8.7)	32.4	35.9
				
Shoulder joint/humerus				
*x*-axis translation	0	0.1 (± 0.1)	0.1	0.2
*y*-axis translation	0	0.0 (± 0.1)	0.0	0.1
*z*-axis translation	0	0.0 (± 0.1)	0.0	0.0
*x*-axis rotation	3.3 (± 7.2)	-20.3 (± 4.6)	23.6	23.8
*y*-axis rotation	2.9 (± 1.2)	-10.4 (± 6.2)	13.3	18.7
*z*-axis rotation	-77.4 (± 11.4)	-117.1 (± 18.2)	39.7	57.7
				
Sterno-clavicular joint/clavicle				
*x*-axis translation	0	-0.3 (± 0.2)	0.3	0.4
*y*-axis translation	0	0.4 (± 0.2)	0.4	0.4
*z*-axis translation	0	-0.4 ± (0.2)	0.4	0.6
*x*-axis rotation	83.8 (± 16.4)	20.6 (± 21.1)	63.2	68.2
*y*-axis rotation	-22.0 (± 2.4)	-9.0 (± 1.3)	13.0	19.6
*z*-axis rotation	-43.7 (± 8.0)	-6.2 (± 7.7)	37.5	43.3

All translations were set to zero at the instant of touch down. Translations are reported in cm, rotations in degrees.

### Three dimensional movements of the scapula

**Figure 5:**
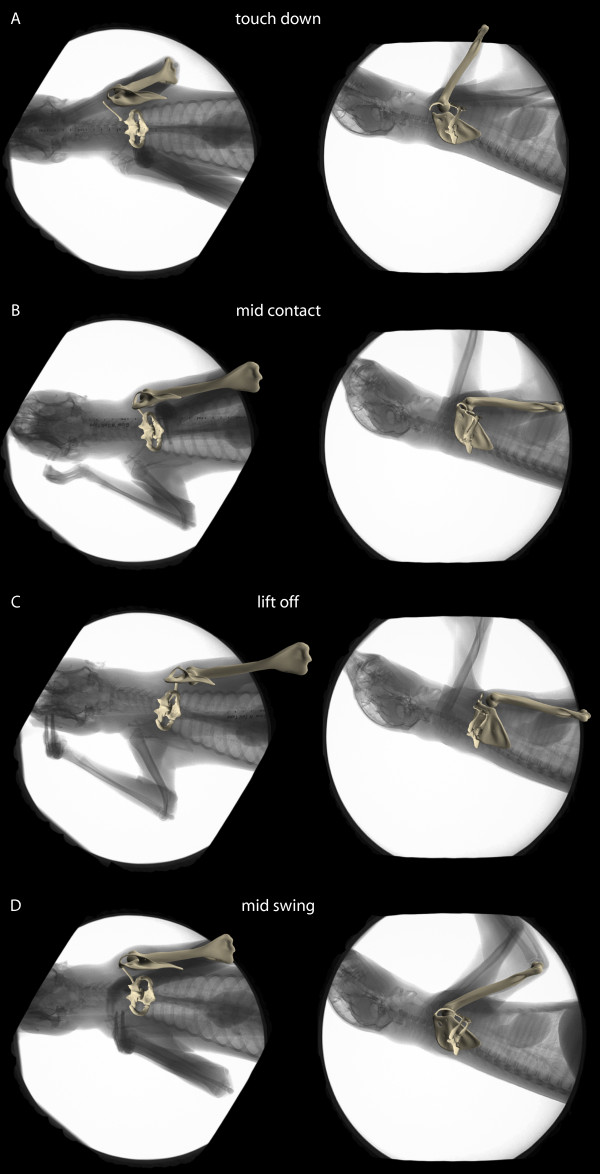
**Representative frames of instants of (A) touch down, (B) mid contact, (C) lift off, and (D) mid swing**. X-ray image with bone model posed to match the x-ray shadow is shown for the dorso-ventral projection and the latero-lateral projection.

Movements of the shoulder blade follow the morphology of the thorax and are confined by the clavicle (Fig. 5). The center of rotation of scapular protraction and retraction is positioned at the vertebral border of the scapula. We documented slight translations of the center of rotation in the second half of the contact phase (Fig. 4B), the most prominent being a caudal translation along the thoracic wall with a maximum of about 0.8 cm on average (Table 4). At the same time the center of rotation is translated laterally and ventrally.

At initial contact the scapula is positioned cranially and dorsally (Fig. 5). It is almost maximally protracted (rotated about the *z*-axis 72° from vertical), rotated inward to about 26° and mean net abduction is -14° (i.e. the shoulder joint is positioned more medial than the scapular center of rotation). After about 30% of the contact phase, scapular retraction, i.e., rotation about the *z*-axis (Fig. 4B), sets in. During retraction the scapula glides around the rounded thorax and the degree of scapular rotation about the long axis decreases to a minimum of about 18° shortly before lift off. The negative abduction, on the other hand, remains relatively constant. At maximum retraction the long axis of the scapula is orientated 39° from vertical (36° maximum amplitude; Table 4). The laterally displaced tapered thorax almost positions the scapula into the parasagittal plane, but it is still rotated inwards (about its long axis) at lift off. The ventro-caudal movement of the glenoid during the contact phase describes a semi-circle, with its distance to the sternum kept relatively constant by the clavicle. At lift-off the glenoid fossa faces ventral. Scapula protraction starts shortly before lift-off and full protraction is reached shortly before the limb touches down again.

### Movements in the gleno-humeral joint

Only minimal translations of less than two millimeters are observed in the gleno-humeral joint (Fig. 4C) - minute motions which are only slightly above our estimated analytical grade of repeatability. During the initial third of the contact phase, when there is little scapula movement, the upper arm is retracted exclusively via flexion in the gleno-humeral joint (rotation about *z*-axis Fig. 4C, Fig. 5). Flexion starts immediately before touch down from maximal extension (approx. -70° from long axis of scapula), and full flexion (approx. -125° from long axis of scapula) is reached shortly after mid contact. In the second half of the contact phase the gleno-humeral joint extends slowly and continues this extension for most of the swing phase until it reaches full extension again. During steady-state locomotion in *C. didactylus*, humeral abduction from the scapular plane is moderate (maximal amplitude during contact approx. 13°).

### Movements of the clavicle

The sternal end of the clavicle translates up to 0.6 cm relative to the manubrium sterni (Table 2, Fig. 4D). These movements are facilitated by the connective tissue making up the sterno-clavicular articulation (SCA) (Fig. 3). At touch down, the clavicle is rotated approximately 84° about its long axis (rotation about *x*-axis in Fig. 4D) and protracted by about 22° (indicated by a negative value for rotation about *y*-axis). The acromio-clavicular joint is positioned more dorsal than the manubrium sterni (44° rotation about *z*-axis). During limb retraction rotation about the long axis decreases to about 20° and the clavicle is both retracted and depressed so that at lift off, the acromio-clavicular joint and the SCA are at the same height. It is noteworthy that caudal displacement of the acromio-clavicular joint is achieved not only by rotation about the *y*-axis, but also by the pronounced rotation about the long axis of the curved clavicle.

### 3D displacement of the elbow: effects of scapular and humeral motion

**Figure 6:**
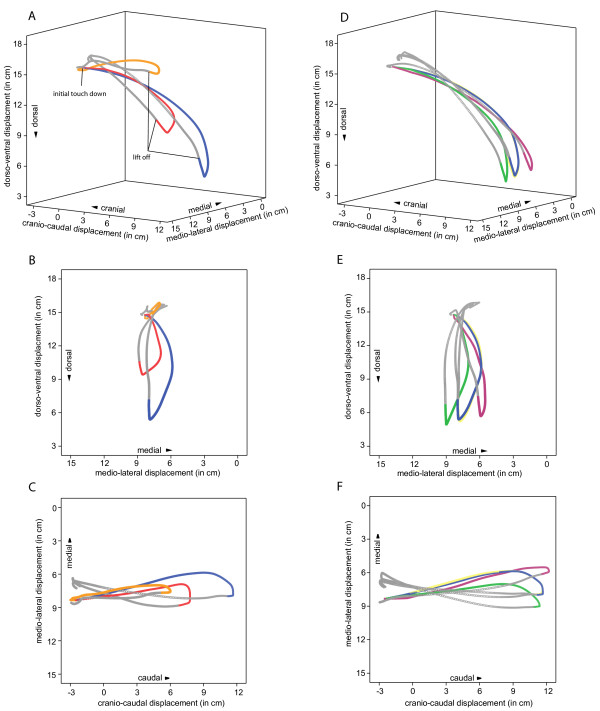
**3D displacement of the right elbow and the influence of scapular and humeral motion**. A, D: 3D trajectories of the elbow shown for normal locomotion (blue), without humeral motion (orange), without scapular motion (red), without scapular abduction/adduction (yellow - almost completely covered by blue trajectory, i.e. almost identical), without scapular rotation along its long axis (green), and without humeral abduction from the scapular plane (purple). Swing phases are shown in gray. B, E: depict a 2D projection of the trajectory onto the transversal plane as seen from behind. C, F: 2-D projection of the trajectory onto the frontal plane as seen from above.

The displacement of the elbow is governed by both scapular and humeral motions, i.e., trajectories of elbow displacement differ markedly if scapular or humeral motion is 'muted' (Fig. 6A, D). Normal locomotion has a medio-lateral amplitude of displacement of the elbow of 2.5 cm relative to the 1^st ^thoracic vertebra (Table 5, Fig. 6B). The amplitude of medio-lateral displacement is just 1.7 cm if all humeral translations and rotations in the gleno-humeral joint are turned off. The amplitude remains at 2.5 if scapular motion is turned off completely in the 'virtual experiment', however both the maximum and the minimum increase. The 'muting' of scapular abduction/adduction has almost no effect on elbow displacement as these movements are rather minor (Table 5; Fig. 6D). The turning off of scapular as well as humeral rotation about their respective long axes both increases the amplitude of medio-lateral displacement of the elbow to 3.1 cm. This means that the rotation about the long axis in the scapula and humeral abduction/adduction partly offset each other and observed medio-lateral displacement of the elbow is smaller (Fig. 6E). In sum, total abduction of the arm is determined by the combination of the scapula's position relative to the rounded rib cage (i.e., scapular rotation about the long axis and scapular protraction and retraction) and humeral abduction from the scapular plane.

**Table 5:** Mean maximal and minimal 3D displacements of the elbow relative to the 1^st ^thoracic vertebra during normal locomotion and 'virtual experiments' to assess the displacing effect of the motion of the scapula and humerus.

	Max	Min	Amplitude	% tfl
I. medio-lateral displacement				
Normal locomotion (mean)	8,3	5,9	2,5	5.1
Without total humeral motion	8,7	7,0	1,7	3.5
Without total scapular motion	8,9	6,4	2,5	5.1
Without scapular abduction/adduction	8,3	5,9	2,5	5.1
Without scapular long-axis rotation	9,1	6,0	3,1	6.3
Without humeral abduction/adduction	8,7	5,5	3,1	6.3
				
II. cranio-caudal displacement				
Normal locomotion (mean)	11,7	-2,8	14,4	29,3
Without total humeral motion	6,0	-3,0	9,0	18.3
Without total scapular motion	7,8	-2,8	10,6	21.5
Without scapular abduction/adduction	11,6	-2,9	14,6	29.7
Without scapular long-axis rotation	11,4	-2,8	14,2	28.9
Without humeral abduction/adduction	12,2	-2,9	15,2	30.1
				
III. dorso-ventral displacement				
Normal locomotion (mean)	15,7	5,4	10,3	20.9
Without total humeral motion	15,9	14,5	1,4	2.8
Without total scapular motion	15,6	9,4	6,2	12.6
Without scapular abduction/adduction	15,7	5,3	10,4	21.1
Without scapular long-axis rotation	15,8	5,0	10,9	22.2
Without humeral abduction/adduction	15,2	5,7	9,4	19.1

All values are reported in cm. Amplitude is also expressed relatively in percent of total forelimb length (% tfl; average length in both individuals: 49.2 cm incl. scapula).

Cranio-caudal displacement of the elbow relative to first thoracic vertebra is predominantly achieved by humeral retraction in the gleno-humeral joint (a decrease in cranio-caudal displacement from 14.4 cm to 9.0 cm if humeral motion is turned off). Scapular retraction also contributes to the amplitude of elbow displacement as the amplitude decreases to 10.6 cm if scapula motion is 'muted' virtually in the 3D animated reconstruction (Fig. 6C). Scapular abduction/adduction and scapular rotation about its long axis has only little influence on the cranio-caudal amplitude of the 3D trajectory of displacement of the elbow (Table 5). Turning off humeral abduction/adduction slightly increases the cranio-caudal amplitude of elbow displacement.

Dorso-ventral displacement of the elbow is largely determined by humeral retraction (Table 5) with only little influence of the scapula. However, as evident in the graph (Fig. 6B) only the combination of both movements effectively displaces the elbow in dorso-ventral direction. Scapular abduction/adduction as well as rotation about its long axis again has very limited influence on the displacement of the elbow. The amplitude of dorso-ventral displacement is slightly reduced if humeral abduction/adduction is 'muted' in our 'virtual experiment' (from 10.3 cm to 9.4 cm).

## Discussion

### Significance of scapular motion

Humeral retraction has been shown to be responsible for over 70% of stride length in two-toed sloths [16]. Accordingly, most of the cranio-caudal displacement of the elbow observed in this study is generated by retraction of the humerus in the gleno-humeral joint. However, despite emphasized influence of humeral motion to the displacement of the hand, the overall pattern of forelimb movement is very similar to pronograde mammals: forelimb movement in sloths takes place at the most proximal pivot possible through the fixing of distal limb joints and retraction of the scapula as the most proximal element [16].

Scapula movement has been demonstrated via x-ray motion analysis to be an important aspect of forelimb kinematics during quadrupedalism in therian mammals [3,5,7,23,24]. The six DOF of scapular motion quantified here correspond to previous qualitative descriptions of three dimensional scapular movement in claviculate arboreal and terrestrial pronograde therian mammals (Table 6), but differ from the 3D data quantified in walking cats and the qualitative description of shoulder movements in brachiating spider monkeys [25,26]. Whereas in the aclaviculate cat there is an effective rotation about the scapular long axis of less than 2° [25], rotation about the long axis of the scapula during the contact phase of sloths has an approximate 8° amplitude (Table 4). The observation of movements that are largely restricted to the parasagittal plane in aclaviculate mammals is also in line with results from an analysis of rats with excised clavicles [23]. But the scapula, as in the cat, remains slightly abducted throughout the stride cycle in the sloth and has only a small amplitude during the contact phase (less than 1°; Table 4). Motions of the shoulder blade during quadrupedal suspensory locomotion of the sloth are also in stark contrast to the motions described for brachiating spider monkeys [26]. Spider monkeys maintain the scapula in a distinct dorsal orientation and the fossa glenoidalis faces cranially throughout the contact of the limb [26]. In similar fashion as in the claviculate pronograde species investigated [27,28], a relatively round thorax permits the scapula in the two-toed sloth to effectively slide dorsally during forelimb protraction from it's almost parasagittal orientation at lift off. This is accomplished via a combination of protraction, long-axis rotation and abduction/adduction. The more dorsal orientation of the scapula at touch down inevitably results in a more laterally facing fossa glenoidalis. The orientation of the scapula at touch down has an abducting effect on the whole forelimb. This indicates that, despite the adoption of obligatory quadrupedal suspensory locomotion, the basic kinematics of the scapula remained practically unchanged. The only striking differences are that scapular retraction sets in later in the contact phase and the initial period is marked by humeral retraction alone in sloths.

**Table 6:** Published data on scapular movements in mammals.

Species	Body mass	Mean amplitude of protraction/retraction during contact phase	Data on 3D motion	References
*Monodelphis domestica *(Didelphidae)	0.05 - 0.075 kg	59°	-	[5]
*Microcebus murinus *(Primates)	0.05 - 0.1 kg	48° ± 6°	Qualitative description	[5,34,43]
*Tupaia glis *(Scandentia)	0.05 - 0.180 kg	59°	-	[5,44]
*Dasyuroides byrnei *(Dasyuridae)	0.1 - 0.12 kg	44°	-	[5]
*Rattus norvegicus *(Rodentia)	0.14 - 0.4 kg	60°	-	[5,23]
*Saguinus Oedipus *(Primates)	0.35 - 0.45 kg	49° ± 6°	Qualitative description	[32,43]
*Galea musteloides *(Rodentia)	0.4 - 0.5 kg	60°	-	[5]
*Cavia porcellus *(Rodentia)	0.6 - 1.0 kg	57° *	-	[45]
*Saimiri scuireus *(Primates)	0.365 - 1.135 kg	55° ± 4°	Qualitative description	[43,46]
*Procavia capensis *(Hyracoidea)	1.8 - 5.4 kg	53°	-	[3,5]
*Eulemur fulvus *(Primates)	≈ 3.0 kg	51° ± 9°	Qualitative description	[34,43,47]
*Didelphis virginiana *(Didelphidae)	2.0 - 5.5 kg	40°	Qualitative description	[28]
*Cercopithecus aethiops *(Primates)	2.5 - 6 kg	28° **	-	[24]
*Felis catus *f. domestica (Carnivora)	3.0 - 8.0 kg	41°	3D movements quantified	[25,48]
*Choloepus didactylus *(Xenarthra)	4.0 - 10.0 kg	32° (34° ***)	3D movements quantified (6 DOF)	[16], this study
*Ateles geoffroyi/Ateles paniscus *(Primates)	7.5 -8.4 kg/7.75 - 9.5 kg	15° ****	Qualitative description	[26]
*Canis lupus *f. familiaris (Carnivora)	15 - 80 kg	35° ± 4°	-	[7]
*Capra hircus *(Artiodactyla)	25 - 70 kg	41° ± 7°	-	[7]
*Equus przewalski *f. caballus (Perissodactyla)	≈ 350 kg	25° ± 5°	-	[7]
*Loxodonta africana *(Proboscidea)	3500 - 7000 kg	15° ± 5°	-	[7]

*:determined from figure 3 in [45]; **: determined from figure 2 in [24]; ***:when projected into the parasagittal plane as in [16]; ****: during brachiation

Quantification of 3D motion is rare. Please note that the scapular protraction and retraction of the two-toed sloth is very similar to quadrupedal mammals of similar weight. All body masses according to [42].

Overall mobility of the shoulder in mammals results always from a combination of mobility in the gleno-humeral joint and mobility of the pectoral girdle, which is determined by the shape of thorax, relative position of the scapula, and configuration of the clavicle [29]. However, mobility may not be confused with in vivo movement during linear locomotion, which should only represent a fracture of overall mobility.

When thorax width is compared to the dataset of different primate taxa analyzed by Kagaya et al. [30], the sloth has a smaller width (log thoracic width 1,63 mm; log body mass 0.93 kg; cf. [30]). The small diameter and rounded shape of the thorax of sloths emphasizes the 3D excursions of the comparably small scapula during forelimb protraction. Based on x-ray motion analysis, abduction generated from scapular movement seems to be emphasized in the investigated small quadrupedal primates that likely resemble the ancestral anatomical condition regarding the shape of the thorax and the position of the scapula [27]. Nevertheless, overall great mobility of the pectoral girdle as well as great mobility in the gleno-humeral joint, probably used in non-locomotor behavior, has been shown for a large dataset of primates [29,31]. The combination of a small scapula and a rounded, small-diameter thorax of sloths represents a solution to the functional demand of extensive forelimb mobility in the arboreal context. Additionally, configuration of the shoulder joint may permit extensive medio-lateral excursions of the limb by abduction of the humerus from the scapular plane as present in most Anthropoidea during locomotion [32]. Although limb abduction during quadrupedal suspensory locomotion of sloths is small, considering the morphological and kinematic data presented here we suggest that in sloths both modes are present (i.e., abduction via scapular movements and via humeral abduction from the scapular plane). Forelimb abduction in early contact phase is accomplished by a combination of an inward rotation of the scapula along its long axis with a simultaneous cranial displacement of the gleno-humeral joint by protraction of scapula. At this time the humerus remains approximately in the scapular plane. During the later contact phase outward rotation of the scapula along its long axis adducts the elbow. This adduction is offset by humeral abduction from the scapular plane, which displaces the elbow lateral (please compare green and purple trajectories in Fig. 6). The resulting trajectory is has a small medio-lateral amplitude. Interestingly, the combination of a small scapula and a rounded thorax is also present in Loridae [33,34], i.e., in other deliberate arboreal species unable to jump and with the need for increased forelimb mobility, especially for bridging, due to the discontinuous nature of their arboreal substrates.

In terrestrial quadruped therian mammals the scapula usually dominates forelimb propulsion and, especially in cursorial species, limb movement is restricted more or less to the parasagittal plane, with the scapula orientated lateral to the thoracic wall [7]. A relatively short scapula on the one hand decreases its effect in displacing distal elements of the limb in cranio-caudal direction, but on the other hand facilitates more pronounced 3D motions along the thoracic wall (e.g., from the lateral, retracted orientation at lift off to the more dorsal, protracted position at touch down). The relatively short scapula thus facilitates displacements of the limb in the medio-lateral direction through 3D displacements of the limb provoked by marked 3D motions relative to the thorax.

In this context it is important to note that the scapula, as a newly propulsive skeletal element added proximally to the forelimb in therian mammals, has an independent developmental program from the serially homologous elements of the fore- and hindlimbs (stylopod, zeugopod, and autopod) [6,35]. Variation of overall configuration and relative proportions of the serially homologous elements in the fore- and hindlimbs might be constrained by shared developmental programs [6], but - thanks to its individual developmental program - morphology of the scapula can probably evolve more freely according to demands that act specifically on the forelimbs. Accordingly, the scapula has been found to be the most variable element in both fore- and hindlimbs [6].

### Clavicular motion

The clavicle guides all scapular movements along the thoracic wall [23]. With the distance between sternum and acromion kept relatively constant by the clavicle, the trajectory of the gleno-humeral joint of the sloth describes an arch during the contact phase of a stride cycle. The same phenomenon has been described qualitatively for other claviculate therian mammals (rat [23]; opossum [28]; small quadrupedal primates [27]). Voisin argued that the internal curvature of the clavicle of gibbons (*Hylobates*) and spider monkeys (*Ateles*) facilitates this bone's function as a crank during arm flexion and helps the glenoid cavity of the scapula to effectively rotate cranially [36]. The author further proposes that internal curvature of the clavicle may be linked to suspensory postures in these primates [36]. The strong internal curvature of the clavicle in sloths supports this notion.

In pronograde mammals the clavicle is thought to function as a 'spoke' and a 'strut' [23] and thus to transmit compressive forces between the limb and the trunk. The causal theory of histogenesis put forward by Pauwels [37] predicts that connective tissue differentiates according to its loading regime. Under compressive loads fibrocartilage differentiates within tendons and ligaments [38]. The absence of fibrocartilage in the SCA in two-toed sloths demonstrates that not only does tensile loading act on the distal limbs [9], but that tension is also transferred to the thorax (Fig. 4). Collagen fibers are differentiated in the SCA of *C. didactylus *in the same way as Pauwels [37] predicts for a tensile loading regime. It is intriguing to hypothesize that ossification remains incomplete at the sternal end of the clavicle (a bone that is part of the dermal skeleton) during ontogeny because of the lack of a specific stimulus (here compressive load) to differentiate an articulating face towards the manubrium sterni. A SCA composed of irregular dense fibrous connective tissue not only provides passive stability against translations and rotations [39] but complies with the added demand for increased pectoral girdle mobility in a discontinuous habitat. The absence of elastic fibers negates the possibility of elastic energy storage, although the sternal end of the clavicle translates as far as 0.8 cm cranial from its lift off position. In armadillos and the tamandua the clavicle does not articulate directly with the manubrium sterni either, and the SCA is made up of fibrous connective tissue here too [40,41]. However, it is not known whether fibrocartilage tissue differentiates as an adaptation to compressive load in these species. A comparative study in Xenarthra would yield further insights into the evolution of this interesting trait.

## Summary and conclusion

The adoption of deliberate, non-agile locomotion within a discontinuous habitat made 3D limb excursions necessary, e.g., to facilitate bridging of gaps between branches or to reach for food. Sloths accomplish increased forelimb mobility through a combination of three morphological specializations, whereas the overall 3D kinematic pattern of the pectoral girdle remains remarkably unchanged, when compared to pronograde quadruped therians with developed clavicles [23,27,28]. Morphological specializations at the pectoral girdle for 3D limb excursions are i) a relatively short scapula in combination with a round, small diameter thorax, ii) maximized mobility at the SCA, and iii) an internally curved clavicle that allows effective cranial displacement of the shoulder.

Results of this study somewhat contradict Miller's [8] notion that sloths may have the strangest mode of progression amongst mammals. In the two-toed sloth morphological specializations facilitate pronounced forelimb mobility necessary in the discontinuous 3D habitat. But, increased forelimb mobility through morphological specialization at the same time allowed the retention of the plesiomorphic kinematic pattern.

## Competing interests

The authors declare that they have no competing interests.

## Authors' contributions

Both authors conceived of the study, designed the experiments, dissected the cadavers, interpreted the data, contributed to, and approved the final manuscript. JAN took care of and trained the animals, visited the museum collections, conducted all experiments, reconstructed bone models from CT scans, performed XROMM analysis, and drafted the manuscript.

## References

[B1] Eaton TH jr Modifications of the shoulder girdle related to reach and stride in mammals J Morph 1944 75 167 171 10.1002/jmor.1050750108

[B2] Kuznetsov AN Comparative functional analysis of the fore- and hindlimbs in mammals Zool J Moscow 1985 64 1862 1867 (in Russian)

[B3] Fischer MS Crouched posture and high fulcrum, a principle in the locomotion of small mammals: the example of the rock hyrax (*Procavia capensis*) (Mammalia: Hyracoidea) J Hum Evol 1994 26 501 524 10.1006/jhev.1994.1030

[B4] Gasc JP Comparative aspects of gait, scaling and mechanics in mammals Comp Biochem Physiol A 2001 131 121 133 10.1016/S1095-6433(01)00457-3 11733171

[B5] Fischer MS Schilling N Schmidt M Haarhaus D Witte H Basic limb kinematics of small therian mammals J Exp Biol 2002 205 1315 1338 11948208 10.1242/jeb.205.9.1315

[B6] Schmidt M Fischer MS Morphological integration in mammalian limb proportions: dissociation between function and development Evolution 2009 63 749 766 10.1111/j.1558-5646.2008.00583.x 19087186

[B7] Fischer MS Blickhan R The tri-segmented limb of therian mammals: kinematics, dynamics, and self-stabilization - a review J Exp Zool 2006 305A 935 952 10.1002/jez.a.333 17029268

[B8] Miller RA Functional adaptations in the forelimb of the sloths J Mammal 1935 16 38 51 10.2307/1374529

[B9] Patel BA Carlson KJ Apparent density patterns in subchondral bone of the sloth and anteater forelimb Biol Lett 2008 4 486 489 10.1098/rsbl.2008.0297 18628113 PMC2610091

[B10] Mendel FC Montgomery GG Adaptations for suspensory behavior in the limbs of two-toed sloths The Evolution and Ecology of Armadillos, Sloths, and Vermilinguas 1985 Washington: Smithonian Institution Press 151 162

[B11] Mendel FC The wrist joint of two-toed sloths and its relevance to brachiating adaptations in the Hominoidea J Morph 1979 162 413 424 10.1002/jmor.1051620308 30213156

[B12] Mendel FC The hand of two-toed sloths (*Choloepus*): its anatomy and potential uses relative to size of support J Morph 1981 169 1 19 10.1002/jmor.1051690102 30139204

[B13] Mendel FC Foot of two-toed sloths: its anatomy and potential uses relative to size of support J Morph 1981 170 357 372 10.1002/jmor.1051700307 30119592

[B14] Mendel FC Use of hands and feet of two-toed sloths (*Choloepus hoffmanni*) during climbing and terrestrial locomotion J Mammal 1981 62 413 421 10.2307/1380728

[B15] Mendel FC Use of hands and feet of three-toed sloths (*Bradypus variegatus*) during climbing and terrestrial locomotion J Mammal 1985 66 359 366 10.2307/1381249

[B16] Nyakatura JA Petrovitch A Fischer MS Limb kinematics during locomotion in the two-toed sloth (*Choloepus didactylus*, Xenarthra) and its implications for the evolution of the sloth locomotor apparatus Zoology in press 10.1016/j.zool.2009.11.003 20637572

[B17] Davis DD The shoulder architecture of bears and other carnivores Fieldiana Zoology 1949 81 285 305

[B18] Carrier DR Deban SM Fischbein T Locomotor function of the pectoral girdle 'muscular sling' in trotting dogs J Exp Biol 2006 209 2224 2237 10.1242/jeb.02236 16709923

[B19] Lucae JCG The muscles and the skeleton of the black lemur and sloth (Lemur macaco and Choloepus didactylus) Frankfurt a. M.: Mahlau und Waldschmidt Verlag, Senckenbergische Naturforschende Gesellschaft 1882 (in German)

[B20] Gatesy SM Baier DB Jenkins FA Dial KP Scientific rotoscoping: a morphology-based method of 3D-motion analysis and visualization J Exp Zool 2010 313A 280 298 10.1002/jez.588 20084664

[B21] Brainerd EL Baier DB Gatesy SM Hedrick TL Metzger KA Crisco JJ X-ray reconstruction of moving morphology (XROMM): Precision, Accuracy and Applications in Comparative Biomechanics research J Exp Zool 2010 313A 262 279 10.1002/jez.589 20095029

[B22] Flower WH On the mutual affinities of the animals composing the order Edentata Proc Zool Soc 1882 50 358 367

[B23] Jenkins FA The movement of the shoulder in claviculate and aclaviculate mammals J Morph 1974 144 71 84 10.1002/jmor.1051440105 4416370

[B24] Whitehead PF Larson SG Shoulder motion during quadrupedal walking in *Cercopithecus aethiops*: integration of cineradiographic and electromyographic data J Hum Evol 1994 26 525 544 10.1006/jhev.1994.1031

[B25] Boczek-Funke A Kuhtz-Buschbeck JP Illert M Kinematic analysis of the cat shoulder girdle during treadmill locomotion: an x-ray study Europ J Neuroscience 1996 8 261 272 10.1111/j.1460-9568.1996.tb01210.x 8714697

[B26] Jenkins FA Dombrowski PJ Gordon EP Analysis of the shoulder in brachiating spider monkeys Am J Phys Anthropol 1978 48 65 76 10.1002/ajpa.1330480110 414626

[B27] Schmidt M Krause C D'Aout K, Vereecke EE Scapula movements and their contribution to three-dimensional forelimb excursions in quadruped primates Primate Locomotion: Linking Field and Laboratory Research New York: Springer in press

[B28] Jenkins FA Weijs WA The functional anatomy of the shoulder in the Virginia Opossum (*Didelphis virginiana*) J Zool (Lond) 1979 188 379 410 10.1111/j.1469-7998.1979.tb03423.x

[B29] Chan LK The range of passive arm circumduction in primates: do hominins really have more mobile shoulders? Am J Phys Anthropol 2008 136 265 277 10.1002/ajpa.20800 18324636

[B30] Kagaya M Ogihara N Morphological study of the anthropoid thoracic cage: scaling of thoracic width and analysis of rib curvature Primates 2008 49 89 99 10.1007/s10329-007-0064-z 17902025

[B31] Chan LK Glenohumeral mobility in primates Folia Primatol 2007 78 1 18 10.1159/000095682 17170553

[B32] Schmidt M Schilling N Fiber type distribution in the shoulder muscles of the tree shrew, the cotton-top tamarin, and the squirrel monkey related to shoulder movements and forelimb loading J Hum Evol 2007 52 401 419 10.1016/j.jhevol.2006.11.005 17289114

[B33] Strauss WL Wislocki GB On certain similarities between sloths and slow lemurs Bull Mus Comp Zool Harvard College 1932 74 45 56

[B34] Schmidt M Forelimb proportions and kinematics: are small primates different from other small mammals? J Exp Biol 2008 211 3775 3789 10.1242/jeb.019802 19043050

[B35] Huang R Zhi Q Patel K Wilting J Christ B Dual origin and segmental organization of the avian scapula Development 2000 127 3789 3794 10934023 10.1242/dev.127.17.3789

[B36] Voisin JL Clavicle, a neglected bone: morphology and relation to arm movements and shoulder architecture in primates Anat Rec 2006 288A 944 953 10.1002/ar.a.20354 16894572

[B37] Pauwels F A new theory for the influence of mechanical stimuli on the differentiation of supporting tissue Anat and Embryol 1960 121 478 515 (in German) 10.1007/BF00523401 14431062

[B38] Benjamin M Ralphs JR Fibrocartilage in tendons and ligaments - an adaptation to compressive load J Anat 1998 193 481 494 10.1046/j.1469-7580.1998.19340481.x 10029181 PMC1467873

[B39] Ralphs JR Benjamin M The joint capsule: structure, composition, aging and disease J Anat 1994 184 503 509 7928639 PMC1259958

[B40] Miles SS The shoulder anatomy of the armadillo J Mammal 1941 22 157 169 10.2307/1374910

[B41] Taylor BK The anatomy of the forelimb in the anteater (*Tamandua*) and its functional implications J Morph 1978 157 347 368 10.1002/jmor.1051570307 30231602

[B42] Apfelbach R Grzimek B Grzimek's encyclopedia of mammals 1997 Leipzig: Brockhaus (in German)

[B43] Schmidt M Voges D Fischer MS Shoulder movements during quadrupedal locomotion in arboreal primates Z Morph Anthrop 2002 83 235 242 12050895

[B44] Schilling N Fischer MS Kinematic analysis of treadmill locomotion of tree shrews, *Tupaia glis *(Scandentia: Tupaiidae) Z Säugetierkunde 1999 64 129 153

[B45] Rocha-Barbosa O Renous S Gasc JP Comparison of the fore and hind limbs kinematics in the symmetrical and asymmetrical gaits of a caviomorph rodent, the domestic guinea pig, *Cavia porcellus *(Linné, 1758) (Rodentia, Caviidae) Annales Sciences naturelles, Zoologie, Paris 1996 17 149 165

[B46] Schmidt M Quadrupedal locomotion in squirrel monkeys (Cebidae: *Saimiri sciureus*) - a cineradiographic study on limb kinematics and related substrate reaction forces Am J Phys Anthropol 2005 128 359 370 10.1002/ajpa.20089 15838834

[B47] Schmidt M Fischer MS Cineradiographic study of forelimb movements during quadrupedal walking in the Brown Lemur (*Eulemur fulvus*, Primates: Lemuridae) Am J Phys Anthropol 2000 111 245 262 10.1002/(SICI)1096-8644(200002)111:2<245::AID-AJPA9>3.0.CO;2-3 10640950

[B48] Miller S van der Meché FGA Movements of the forelimbs of the cat during stepping on a treadmill Brain Research 1975 91 255 169 10.1016/0006-8993(75)90546-6 1164673

